# Causal relationships between telomere length and liver disease: a Mendelian randomization study

**DOI:** 10.3389/fgene.2023.1164024

**Published:** 2023-07-31

**Authors:** Shuangjing Zhu, Mengtao Yang, Ting Wang, Zhen Ding

**Affiliations:** Department of Hepatobiliary Surgery, Chaohu Hospital of Anhui Medical University, Hefei, China

**Keywords:** telomere, cirrhosis, Mendelian randomization, liver, single-nucleotide polymorphisms

## Abstract

**Background:** Leukocyte telomere length and hepatic disorders have been linked in various research studies, although their causative association has not been clarified. This study investigated the causal relationship between the length of telomeres on peripheral blood leukocytes and certain liver disorders.

**Methods:** Mendelian randomization (MR) analysis was used to examine the relationship between leukocyte telomere length and risk of liver disease using the publicly accessible worldwide gene-wide association study (GWAS) database. The weighted mode, weighted median, and inverse variance weighted (IVW) methods were employed as supplements to the IVW approach, which is the main analytical method.

**Results:** Leukocytes with longer telomeres may have a lower risk of developing cirrhosis [OR = 0.645 (0.524, 0.795), *p* = 3.977E-05] and a higher chance of developing benign liver tumors [OR = 3.087 (1.721, 5.539), *p* = 1.567E-04]. There was no direct link between telomere length and fatty liver, hepatic fibrosis, or liver cancer. Our findings in the replication analysis agreed with those of the previous studies.

**Conclusion:** Further research is needed to examine the mechanisms underlying the probable causal association between the length of leukocyte telomeres and cirrhosis and benign liver cancer.

## 1 Introduction

Telomeres are DNA–protein complexes present at the ends of eukaryotic linear chromosomes that protect the genome from loss of genetic material, maintain chromosomal integrity, and regulate the cell division cycle. However, owing to the cell’s replication mechanism, the cell does not entirely reproduce the chromosome, and each division loses 50–100 nucleotides, causing telomeres to gradually shorten, which is known as a physiological marker of cellular and biological aging (Blackburn, 1991; Blackburn et al., 2015; Vaiserman and Krasnienkov, 2020). Previous cross-sectional studies have linked leukocyte telomere length to smoking, obesity, and a lack of exercise (Mirabello et al., 2009; Zhang et al., 2021). Telomere length is also linked to cancer, cirrhosis, liver disease, cardiovascular disease, and other illnesses, according to some research (Mirabello et al., 2009; Carulli and Anzivino, 2014; Mwasongwe et al., 2017; Samavat et al., 2021; Korkiakoski et al., 2022).

One of the key organs in the body that metabolizes primarily in individuals is the liver. Liver damage leads to the manifestation of some common clinical disorders, such as fatty liver, cirrhosis, liver fiber, malignant tumors, and other liver diseases. Although there have been significant advancements in the treatment of liver illnesses, our top objective continues to be the investigation of potential therapies. Telomere length and liver disease are causally related, according to recent studies. According to a cross-sectional study, leukocyte telomere length is independently linked to a high risk of fatty liver, and individuals with shorter telomeres may have more liver fibrosis (Korkiakoski et al., 2022). Leukocyte telomere shortening was independently correlated with patients acquiring liver disease, according to a follow-up study of leukocyte telomere length in 7,072 individuals in the United States (Rattan et al., 2022). However, in a case–control research study, Liu et al. (2011) revealed that individuals with long telomeres were more likely to develop cirrhosis, hepatocellular carcinoma, and persistent HBV infection. Additionally, a nested case–control study supported Liu et al.'s results that the length of leukocyte telomeres in blood or serum was a risk factor for cirrhosis, chronic HBV infection, and even one of the risk factors for individuals with liver cancer (Wan et al., 2012). As a result, we are still unsure of how liver disease and leukocyte telomere length are causally related.

Probably, variables, including the sample source, variety of research individuals, and selection bias, led to the conflicting outcomes of the current study. The Mendelian randomization (MR) technique effectively dealt with the confounding effects of race, sample size, and reverse causality that were present in earlier observational and cross-sectional investigations. Similar to randomized controlled trials, MR examines the association between phenotype and disease using genetic variations as exposures. According to Mendelian’s second law of inheritance, alleles are distributed randomly during gamete formation. While avoiding bias in reverse causal linkages and errors brought on by confounding factors, it explores the causality between phenotype and disease using the diversity of genes as an instrumental variable (Davey Smith and Hemani, 2014). This project used MR analysis to investigate the causal link between genetic variation associated with leukocyte telomere length and fatty liver (NAFLD), cirrhosis, liver fibrosis, benign liver tumors, and hepatocellular cancer. We also performed a replication analysis to support the results of our experiments. According to the literature searches performed so far, MR approaches have not been used to investigate the relationship between peripheral blood leukocyte telomere length and liver disease.

## 2 Materials and methods

Using the most recent gene-wide association study (GWAS) pooled data, the current study conducted the largest two-sample MR analysis to evaluate the causal connection between telomeres and liver disease. Three hypotheses have to be satisfied using the MR analysis (Bowden et al., 2017; Davies et al., 2018): 1) exposure must be substantially correlated with genetic variation; 2) results and any confounding variables connected with them should not be correlated with genetic variations; and 3) genetic variations must only be connected to results through exposure. Our investigation made use of GWAS pooled data that were made accessible to the public, and all relevant staff participating in the study received ethical permission from their individual medical ethics committees. All study participants provided their informed consent as well.

### 2.1 Telomere length data source

We utilized the open GWAS (https://gwas.mrcieu.ac.uk/) database, the largest GWAS database of telomere length genetic variation related to telomeres to date. The database consists of a sizable population-based cohort that the UK Biobank (RRID: SCR_012815) put together from 2006 to 2010, with participants between the ages of 40 and 69. These individuals were thoroughly profiled through questionnaires, physical examinations, plasma biomarkers, genome-wide analyses, and other studies. In their analysis of 489,092 peripheral blood leukocyte DNA samples obtained from UKB, Codd et al. (2022) eliminated samples from study dropouts, duplicates, erroneous leukocyte telomere length measurements, and self-reported and genetic gender inconsistencies. Finally, the measurement data of the telomere length of peripheral blood leukocytes from 472,174 individuals were obtained (Sudlow et al., 2015). We identified relevant SNPs that were highly linked with telomere length from this GWAS pooled data (*p* < 5 × 10^−8^), and the threshold was set at *R* ^2^ < 0.001 and a base pair window size of 10,000 kb to prevent the influence of linkage disequilibrium (LD). When screened single-nucleotide polymorphisms (SNPs) were not discovered in the outcome, they were disregarded. Finally, when matching SNPs in exposure to the result, we removed echo SNPs with intermediate allele frequencies. Based on the aforementioned inclusion and exclusion criteria, the final SNPs discovered were used as instrumental factors. In addition, to validate our conclusions, we extracted SNPs significantly associated with telomere length from a GWAS meta-analysis of 78,592 participants of European ancestry as our replication stage (Li et al., 2020).

### 2.2 Results from the data source

The summary data for outcomes were gathered from the large-scale GWAS pooled data downloaded from the FinnGen (RRID: SCR_022254) database (Table 1). The FinnGen database contains a large number of cohorts drawn from both population-based cohorts and medical biobank samples. Several databases, such as the Finnish Cancer Registry (FCR), use unique national personal identity numbers to correlate genotypes with particular data. Together with accurate and thorough carcinoma data for solid malignancy, the advantages of comprehensiveness, effectiveness, and timeliness are provided (Leinonen et al., 2017; Mars et al., 2020). The majority of our study’s exposures and outcomes were from European populations, with no substantial sample overlap in GWAS populations.

**TABLE 1:** Outcome sample size and source.

Disease	Cases	Controls	Population	Data source
NAFLD	1,098	340,591	European	https://r8.finngen.fi/
Liver fibrosis	130	338,951	European	https://r8.finngen.fi/
Cirrhosis	3,548	338,951	European	https://r8.finngen.fi/
Benign neoplasm: liver	429	342,070	European	https://r8.finngen.fi/
Hepatoma	648	259,583	European	https://r8.finngen.fi/

### 2.3 MR analysis

By mimicking random assignment in an RCT research study, we used a two-way MR analysis technique to evaluate the causal impact of exposure on outcome. The fundamental approach to MR analysis methods is the IVW (inverse variance weighted) random effects method. The IVW technique is fitted using regression without an intercept term and the inverse of the ending variance (quadratic of SE) as a weight. To verify the reliability of the IVW approach, we also used the weighted mode, the MR-Egger method, the weighted median method, and the MR-PRESSO approach to examine pleiotropy and probable outliers (Burgess et al., 2013; Bowden et al., 2016; Burgess et al., 2017; Burgess and Thompson, 2017).

### 2.4 Sensitivity analysis

The MR-Egger approach uses an intercept term to assess the size of horizontal multiplicity; when the *p*-value of the intercept term is substantial, horizontal multiplicity is unavoidable (Burgess and Thompson, 2017). The MR-PRESSO approach may discover outliers for pleiotropic SNPs and offer causal estimates when pleiotropic SNPs are removed (Verbanck et al., 2018). Heterogeneity in causal estimates among all SNPs in the IVW random effects may be assessed using Cochran’s Q test, with a *p*-value larger than 0.05 showing the lack of heterogeneity, making the study’s conclusions more credible. We also employed the leave-one-out technique to investigate the effect of a single SNP on dual-sample MR data. The MR results of the remaining instrument variables are calculated by deleting one instrument variable at a time. If the MR results change significantly after deleting the tool variables, the SNP may be closely related to the results (Li et al., 2021). In addition, we calculated the F-statistic for each instrumental variable and excluded it when the F-statistic value of the instrumental variable was less than 10 (Burgess et al., 2011). All data analyses were carried out in RStudio (RRID: SCR_000432) software 4.2.2, using the “TwoSampleMR” package (version 0.5.6) and the “MR-PRESSO” package (version 0.5.6) (Hemani et al., 2018).

## 3 Results

**FIGURE 1:**
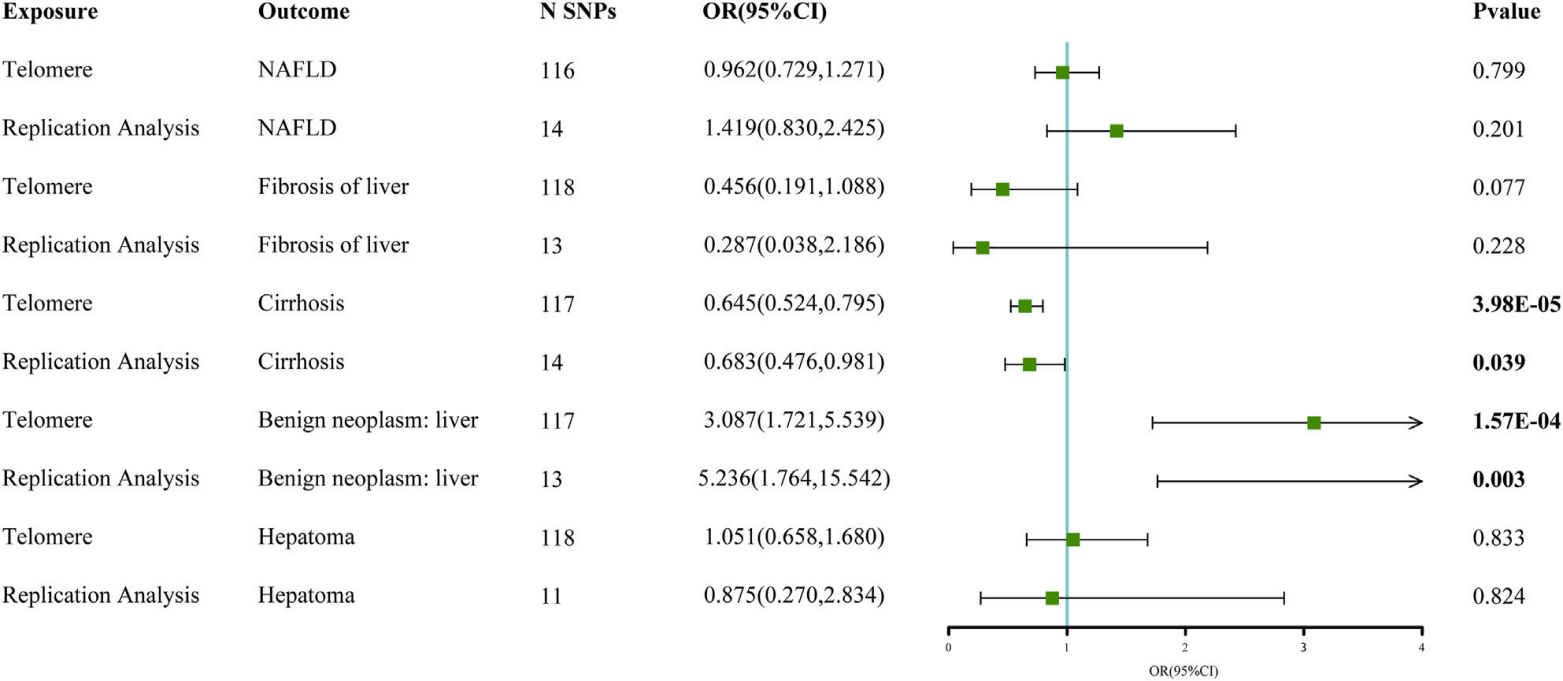
Forest map of telomere and liver disease MR analysis results.

**FIGURE 2:**
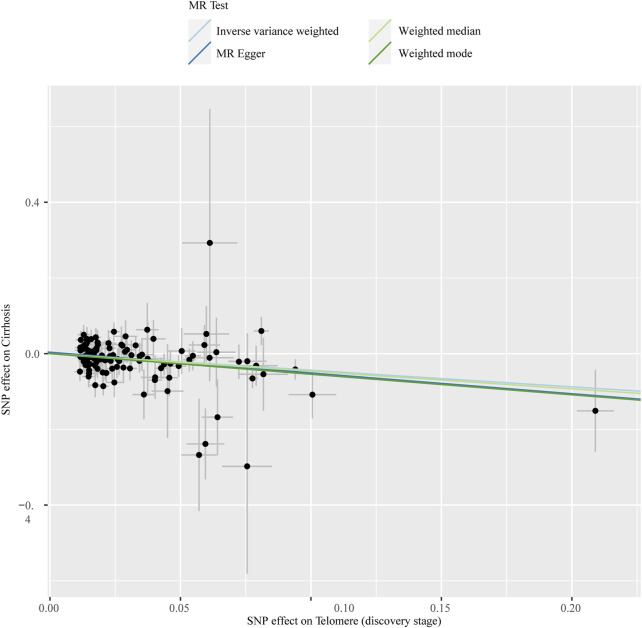
Scatter plot showing the relationship between telomere length and risk of cirrhosis at the discovery stage.

**FIGURE 3:**
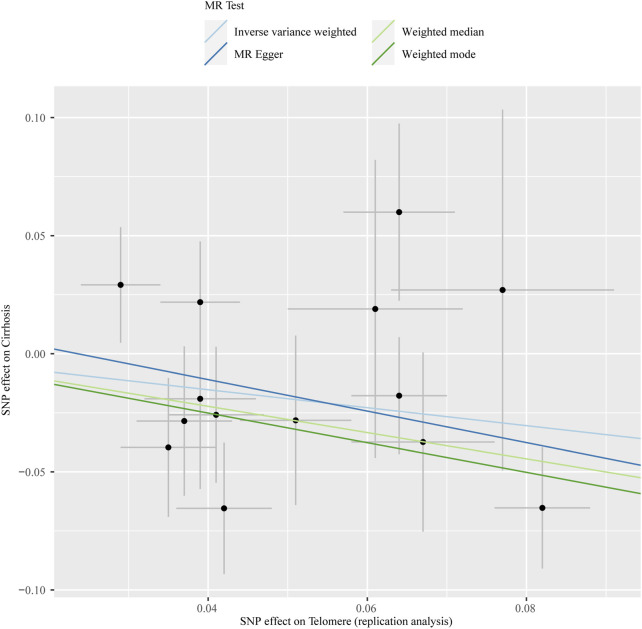
Scatter plot showing the relationship between telomere length and risk of cirrhosis at the replication stage.

**FIGURE 4:**
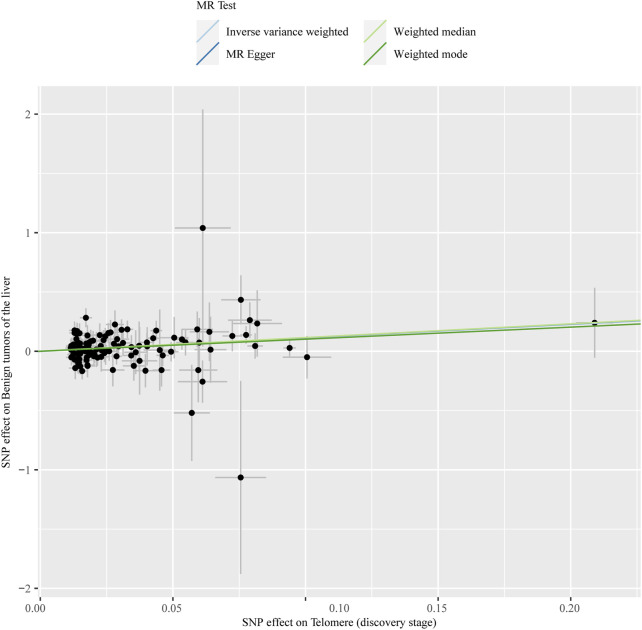
Scatter plot showing the relationship between telomere length and benign liver tumor at the discovery stage.

**FIGURE 5:**
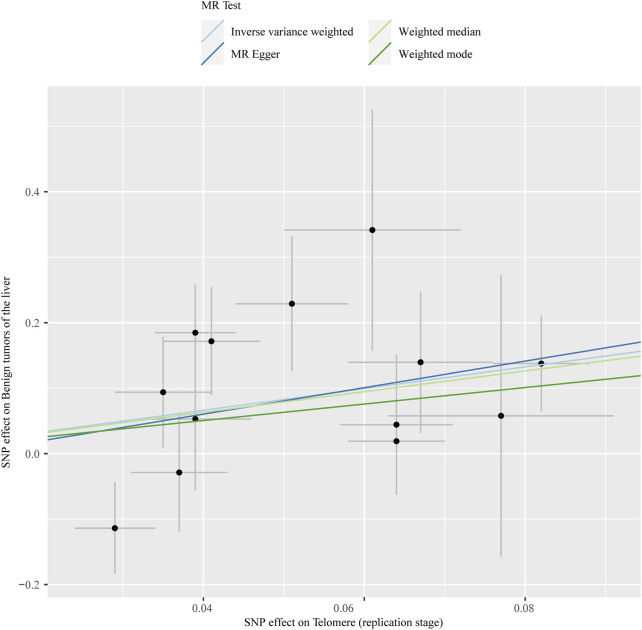
Scatter plot showing the relationship between telomere length and liver benign tumor at the replication stage.

The chosen screening conditions evaluated 118 SNPs for the outcome of liver fibrosis and hepatocellular carcinoma and 117 SNPs for the outcome of fatty liver, cirrhosis, and benign liver tumors. Similar to the original analysis, the replication analysis evaluated 13 SNPs for liver fibrosis, benign liver tumors, and hepatocellular carcinoma and 14 SNPs for fatty liver and cirrhosis. The F-values of these screened instrumental variables were all larger than 10, and there was no bias toward subpar instrumental variables (Supplementary Tables S1, S2). The correlation coefficient between each SNP during exposure and outcome is shown in Supplementary Tables S3, S4. Telomere length may be a protective factor for cirrhosis [OR = 0.645 (0.524, 0.795), *p* = 3.977E-05], a risk factor for benign liver tumors [OR = 3.087 (1.721, 5.539), *p* = 1.57E-04], and not causally associated with fatty liver (*p* = 0.653), liver fibrosis (*p* = 0.077), or liver cancer (*p* = 0.833), according to the current study (Supplementary Table S5). In the replication stage, we discovered comparable results (Figure 1, Supplementary Table S6). In addition, the scatter diagram also shows the relationship between telomere and liver cirrhosis and telomere and benign liver tumor (Figures 2–5). With the use of Cochran’s Q test and the MR-Egger intercept, heterogeneity, and horizontal pleiotropy in MR analysis were discovered. When fatty liver was the result, we discovered heterogeneity (*p* = 0.033). When hepatocellular cancer in the replication stage was the outcome, there was significant heterogeneity (*p* = 0.002) and pleiotropy (*p* = 0.034). The possible SNPs that may alter causation were eliminated using the leave-one-out approach and the MR-PRESSO program; however, the secondary MR analysis did not modify our result (Supplementary Tables S7).

## 4 Discussion

To the best of our knowledge, the current study is the first to examine the causal connection between telomere length and liver illness using MR analysis. In this study, we discovered that, independently of fatty liver, liver fibrosis, and liver cancer, long telomeres may be able to lower the risk of cirrhosis and increase the chance of benign liver tumors. This is comparable to several previous investigations (Calado et al., 2011; Hartmann et al., 2011; Schneider et al., 2022). The study’s conclusions are also strengthened by the heterogeneity and several validity checks of the MR analysis. Our research is based on the analysis of peripheral blood leukocytes, which provide a more accessible method for measuring telomere length in comparison to other tissue types. At present, there is little comparison between the telomere length of peripheral blood leukocytes and other types of human tissues. Demanelis et al. (2020) analyzed telomere length in over 20 different types of human tissues. The results of this study suggest that telomere length in peripheral blood can be indicative of telomere length in many other tissues, thus confirming previous research on the link between telomere length and aging. However, further research is still needed to compare telomere length in whole-blood leukocytes to other human tissues.

A case–control analysis of 134 cirrhotic patients by Calado et al. (2011) revealed that mutations in telomerase, a genetic risk factor for cirrhosis in humans, may cause environmental damage in subjects. This finding further suggests that leukocyte telomere dysfunction is a risk factor for the development of cirrhosis. Hartmann et al. (2011) sequenced the telomerase RNA fraction (TERC) and telomerase reverse transcriptase (TERT) in 521 cirrhotic patients and 600 healthy individuals, indicating that telomerase gene mutations are a risk factor for the development of chronic liver disease into cirrhosis. This confirms that telomerase mutations induce the progression of chronic liver disease toward liver fibrosis and accelerate the formation of chronic liver disease leading to cirrhosis. These studies back up the results of our research.

In contrast to our findings, which did not reveal a correlation between telomere length and fatty liver, liver fibrosis, or liver cancer, other research has shown that leukocyte telomere length may be linked to fatty liver, liver fibrosis, and liver cancer (Liu et al., 2011; Liu et al., 2012; Wojcicki et al., 2017; Kim et al., 2018). Confounding elements like sample size and confounding bias may be accountable for this. Notably, we found a correlation between telomere length and benign liver tumors, which may be due to telomere elongation reducing cellular senescence and promoting the growth and progression of benign tumors. It is worth noting that telomere length is associated with benign liver tumors, which may be due to telomere elongation reducing cell aging and thereby promoting the occurrence and development of benign tumors. A telomere length test was conducted on 13 surviving POT1 mutation carriers from five families, and it was found that gene POT1 mutations prevent telomere shortening and increase the incidence of benign and malignant tumors. This may be due to the loss of the tumor inhibitory mechanism of telomere shortening, leading to the growth of tumor cells (DeBoy et al., 2023). However, this may not fully explain the relationship between white blood cell telomeres and benign liver tumors, and much research is needed in the future to elucidate the underlying mechanism.

Our research offers various advantages. First, our work is based on a large-scale GWAS database with large sample size and an F-statistic value of more than 10 for a selected instrumental variable. Second, we ruled out confounding variables and reverse causality. Finally, to check the stability of our findings, we performed sensitivity analyses such as heterogeneity, pleiotropy, and the leave-one-out approach. At the same time, our research has certain limitations. First, the great majority of the people chosen for this study were of European descent; therefore, our findings cannot be generalized to all races. Second, the instrumental variable telomere length used in this investigation was acquired from blood rather than hepatocyte telomere length. Third, we cannot be certain that all SNPs chosen meet the three fundamental assumptions of the MR analysis. Fourth, although our results suggest an association between leukocyte telomere length and liver disease, because of limitations, additional studies are warranted in the future to corroborate our findings.

## 5 Conclusion

In summary, this study raises the possibility of a causal link between the length of leukocyte telomeres and cirrhosis and benign liver tumors. To corroborate our findings and elucidate the pathophysiological relationships between leukocyte telomere length and liver disease, additional research on other populations is required.

## Data Availability

The original contributions presented in the study are included in the article/Supplementary Material; further inquiries can be directed to the corresponding authors.
